# MDR-1 and MRP-1 activity in peripheral blood leukocytes of rheumatoid arthritis patients

**DOI:** 10.1186/s13000-015-0447-1

**Published:** 2015-12-30

**Authors:** Tamás Micsik, András Lőrincz, János Gál, Richard Schwab, István Peták

**Affiliations:** 1st Department of Pathology and Experimental Cancer Research, Semmelweis University, Budapest, Hungary; Rational Drug Design Laboratories CRC, Semmelweis University, Budapest, Hungary; Department of Rheumatology, Bács-Kiskun County Hospital, Kecskemét, Budapest, Hungary; KPS Medical Biotechnology and Healthcare Services Ltd, Budapest, Hungary; Department of Medical Chemistry and Pathobiochemistry, Pathobiochemistry Research Group of the Hungarian Academy of Sciences, Semmelweis University, Budapest, Hungary; Institute Of Materials And Environmental Chemistry,Research Centre for Natural Sciences, Biological Nanochemistry Research Group, Hungarian Academy of Sciences, 1117 Budapest, Magyar tudósok körútja 2. 1519, P.O. Box 286 Budapest, Hungary

**Keywords:** ABC-transporter, Disease modifying anti rheumatic drugs-DMARDs, Methotrexate, MultiDrug resistance, Rheumatoid arthritis

## Abstract

**Background:**

Rheumatoid Arthritis is a chronic disease leading to decreased quality of life with a rather variable response rate to Disease Modifying Anti Rheumatic Drugs. Methotrexate (MTX) is the gold standard therapy in Rheumatoid Arthritis. The Multidrug resistance Related Protein and Multi Drug Resistance protein 1, also called P-glycoprotein-170 transporters can alter the intracellular concentration of different drugs. Methotrexate is an MRP1 substrate and thus the functional activity of MRP1 might have a clinical impact on the efficiency of the Methotrexate-therapy in Rheumatoid Arthritis.

**Methods:**

We have compared the functional Multidrug Activity Factors (MAF) of the MDR1 and MRP1 transporters of Peripheral Blood Leukocytes of 59 Rheumatoid Arthritis patients with various response rate to MTX-therapy (MTX-responder, MTX-resistant and MTX-intolerant RA-groups) and 47 non-RA controls in six different leukocyte subpopulations (neutrophil leukocytes, monocytes, lymphocytes, CD4+, CD8+ and CD19+ cells). There was a decreased MAF of RA patients compared to non- Rheumatoid Arthritis patients and healthy controls in the leukocyte subpopulations. There was a significant difference between the MAF values of the MTX-responder and MTX intolerant groups. But we have not found significant differences between the MAF values of the MTX-responder and MTX-resistant Rheumatoid Arthritis -groups.

**Results:**

Our results suggest that MDR1 and MRP1 functional activity does not seem to affect the response rate to MTX-therapy of Rheumatoid Arthritis-patients, but it might be useful in predicting MTX-side effects. We have demonstrated the decreased functional MDR-activity on almost 60 Rheumatoid Arthritis patients, which can be interpreted as a sign of the immune-suppressive effect of the MTX-treatment.

## Background

Rheumatoid arthritis (RA) is a chronic inflammatory disease of small articulations often leading to functional impairments and seriously affecting the quality of life [1]. Currently methotrexate (MTX) is the gold standard therapy of the disease modifying antirheumatic drugs (DMARDs), however the response rate is rather variable and many patients are completely resistant to MTX therapy [2, 3]. It has been previously reported, that the Multidrug Resistance Protein 1 (MDR1) and the Multidrug resistance Related Protein 1 (MRP1) transporters - responsible for chemo-resistance of tumor cells - may also have pathophysiological role in different chronic diseases, including rheumatoid arthritis [4, 5].

ATP Binding Cassette (ABC) -transporters are ATP-driven efflux pumps of the cell membranes present on many cell surfaces, but especially on barriers, bile-transporters, kidney-tubules, enterocytes, blood–brain barriers and inflammatory cells. These transporters extrude xenobiotic substances including various drugs via active transport [6]. According to their wide substrate specificity, these transporters may interfere with the absorption and distribution of many therapeutic agents and could influence their serum concentration which in turn can alter the efficiency of different treatments.

The MRP1-transporter substrate MTX is the most widely used DMARD. Therefore, the elevated MDR1/MRP1 expression in the synovium of RA-patients suggested prognostic role to these proteins [3, 7–10]. On the other hand, many studies have reported, that expression levels of MDR1 and MRP1 proteins do not correlate with the functional activity of these proteins predicting the superiority of functional determination of transport activities over expression data [11–13].

The clinical relevance and usefulness of calcein-AM based functional multidrug-assay has been demonstrated in acute myeloid leukemia [14]. We have optimized this assay for the determination of functional MDR1- and MRP1-activity of different peripheral blood leukocyte subtypes of RA-patients to study their possible role in the response to DMARD-therapy.

## Results

Our results suggest that MDR1 and MRP1 functional activity does not seem to affect the response rate to MTX-therapy of Rheumatoid Arthritis-patients, but it might be useful in predicting MTX-side effects. We have demonstrated the decreased functional MDR-activity on almost 60 Rheumatoid Arthritis patients, which can be interpreted as a sign of the immune-suppressive effect of the MTX-treatment. First, we have compared the MAF_Total_, MAF_MDR1_ and MAF_MRP1_ of all six leukocyte subgroups of the five different patient groups to define if there is any significant difference between any of those. We did not find significant differences in the MAF-values of the RA-patients according to their responder-rate to MTX-therapy. Because most of the patients were taking other drugs as well, we have collected the data of medicine-intake with doses and analyzed the potential effect of these on each MAF value. After adjusting the model with the significant drug-effects on the MAF-values, we have compared each RA and non-RA groups pairwise with each other (Table 1).

**Table 1:** Shows the significant differences between different patient groups with (**adjusted p**) and without (**p**) the adjustment of the medicine effects on the different MAF values

Cell type	MAF type	Group 1	Mean	Group 2	Mean	p	Adjusted p	After adjustment
Granulocytes	Total	MTX-intolerant	15	nonRA control	11	**0,0068**	**0,0026**	significant
Granulocytes	Total	MTX-responder	14	nonRA control	11	**0,0097**	0,2005	non significant
Granulocytes	MRP1	MTX-intolerant	5	MTX-responder	2,5	0,3664	**0,0276**	significant
Granulocytes	MRP1	MTX-intolerant	5	nonRA control	−2,5	0,0897	**0,0472**	significant
Granulocytes	MDR1	MTX-resistant	11	MTX-responder	10	0,2475	**0,0284**	significant
Monocytes	Total	MTX-responder	7	nonRA control	12	**0,0214**	0,1114	non significant
Monocytes	Total	MTX-responder	7	Healthy control	15,98	**0,0023**	0,6893	non significant
Monocytes	Total	MTX-intolerant	9	Healthy control	15,98	**0,0266**	0,9483	non significant
Monocytes	Total	MTX-resistant	7,5	Healthy control	15,98	**0,0075**	0,7462	non significant
Monocytes	MDR1	MTX-resistant	7	Healthy control	11,99	**0,0044**	0,6792	non significant
Monocytes	MDR1	MTX-resistant	7	nonRA control	12,5	**0,0245**	0,4869	non significant
Monocytes	MDR1	MTX-intolerant	7	Healthy control	11,99	**0,0071**	0,3590	non significant
Monocytes	MDR1	MTX-responder	5,5	MTX-intolerant	7	0,8766	**0,0276**	significant
Monocytes	MDR1	MTX-intolerant	7	nonRA control	12,5	**0,0443**	**0,0472**	significant
Lymphocytes	Total	Healthy control	34,29	MTX-resistant	27,5	**0,0023**	0,0504	trend
Lymphocytes	Total	Healthy control	34,29	MTX-intolerant	26	**0,0014**	**0,0121**	significant
Lymphocytes	Total	Healthy control	34,29	nonRA control	29	**0,0359**	0,0537	trend
Lymphocytes	Total	Healthy control	34,29	MTX-responder	26	**0,0011**	**0,0084**	significant
Lymphocytes	MRP1	nonRA control	0	MTX-responder	6,5	0,0842	0,0510	trend
Lymphocytes	MRP1	nonRA control	0	MTX-intolerant	7	0,0532	**0,0139**	significant
Lymphocytes	MRP1	nonRA control	0	MTX-resistant	4	0.0689	**0,0022**	significant
Lymphocytes	MRP1	nonRA control	0	Healthy control	10,55	**0,0023**	**0,0014**	significant
Lymphocytes	MDR1	nonRA control	32	Healthy control	21,73	**0,0129**	0,0847	trend
Lymphocytes	MDR1	nonRA control	32	MTX-responder	22,5	**0,0164**	**0,0084**	significant
Lymphocytes	MDR1	nonRA control	32	MTX-resistant	21,5	**0,003**	**0,0008**	significant
Lymphocytes	MDR1	nonRA control	32	MTX-intolerant	21	**0,002**	**0,0007**	significant
CD4+ cells	Total	nonRA control	23,5	MTX-resistant	20	**0,0156**	**0,0270**	significant
CD4+ cells	Total	Healthy control	26,81	MTX-responder	21,5	**0,036**	0,2212	non significant
CD4+ cells	Total	nonRA control	23,5	MTX-intolerant	21	**0,0211**	**0,0064**	significant
CD4+ cells	Total	Healthy control	26,81	MTX-resistant	20	**0,0174**	**0,0278**	significant
CD4+ cells	Total	Healthy control	26,81	MTX-intolerant	21	**0,0032**	**0,0081**	significant
CD4+ cells	MRP1	nonRA control	−1	Healthy control	7,8	**0,0118**	**0,0103**	significant
CD4+ cells	MRP1	Healthy control	7,8	MTX-resistant	2	0,076	**0,0492**	significant
CD4+ cells	MRP1	nonRA control	−1	MTX-intolerant	4	**0,0121**	0,0950	trend
CD4+ cells	MDR1	nonRA control	27,5	MTX-intolerant	16	**0,0004**	**0,0015**	significant
CD4+ cells	MDR1	nonRA control	27,5	MTX-resistant	17,5	**0,0046**	**0,0154**	significant
CD4+ cells	MDR1	nonRA control	27,5	MTX-responder	17	**0,0364**	0,0698	trend
CD4+ cells	MDR1	nonRA control	27,5	Healthy control	17,7	**0,0108**	**0,0448**	significant
CD8+ cells	Total	nonRA control	35	MTX-intolerant	31	**0,0262**	0,0585	trend
CD8+ cells	Total	nonRA control	35	MTX-resistant	31	**0,0078**	**0,0046**	significant
CD8+ cells	Total	nonRA control	35	MTX-responder	29	**0,0008**	**0,0001**	significant
CD8+ cells	Total	Healthy control	40,15	MTX-intolerant	31	**0,0036**	**0,0120**	significant
CD8+ cells	Total	Healthy control	40,15	MTX-resistant	31	**0,0004**	**0,0032**	significant
CD8+ cells	Total	Healthy control	40,15	MTX-responder	29	**0,0002**	**0,0003**	significant
CD8+ cells	Total	MTX-responder	29	MTX-intolerant	31	0,1309	0,0601	trend
CD8+ cells	MRP1	Healthy control	14,35	MTX-intolerant	5	**0,0003**	**0,0004**	significant
CD8+ cells	MRP1	Healthy control	14,35	MTX-resistant	6	**0,002**	**0,0017**	significant
CD8+ cells	MRP1	Healthy control	14,35	MTX-responder	6,5	**0,0069**	**0,0031**	significant
CD8+ cells	MRP1	Healthy control	14,35	nonRA control	0	**0,0053**	**0,0131**	significant
CD8+ cells	MDR1	nonRA control	37,5	MTX-intolerant	28	**0,0302**	0,0634	trend
CD8+ cells	MDR1	nonRA control	37,5	MTX-resistant	24,5	**0,0071**	**0,0014**	significant
CD8+ cells	MDR1	nonRA control	37,5	MTX-responder	21,5	**0,0057**	**0,0012**	significant
CD8+ cells	MDR1	nonRA control	37,5	Healthy control	21,93	**0,0089**	**0,0324**	significant
CD19+ cells	Total	nonRA control	20,5	MTX-resistant	16,5	**0,0435**	0,6173	non significant
CD19+ cells	MRP1	nonRA control	0	MTX-intolerant	5	**0,0426**	**0,0057**	significant
CD19+ cells	MRP1	nonRA control	0	MTX-resistant	3,5	0,3154	**0,0180**	significant
CD19+ cells	MDR1	nonRA control	22,5	MTX-intolerant	14	**0,0175**	**0,0491**	significant
CD19+ cells	MDR1	nonRA control	22,5	MTX-resistant	15	**0,0109**	**0,0000**	significant
CD19+ cells	MDR1	MTX-responder	17	MTX-intolerant	14	0,4548	**0,0058**	significant

Significant *p*-values are highlighted in **bold**

**Fig. 1:**
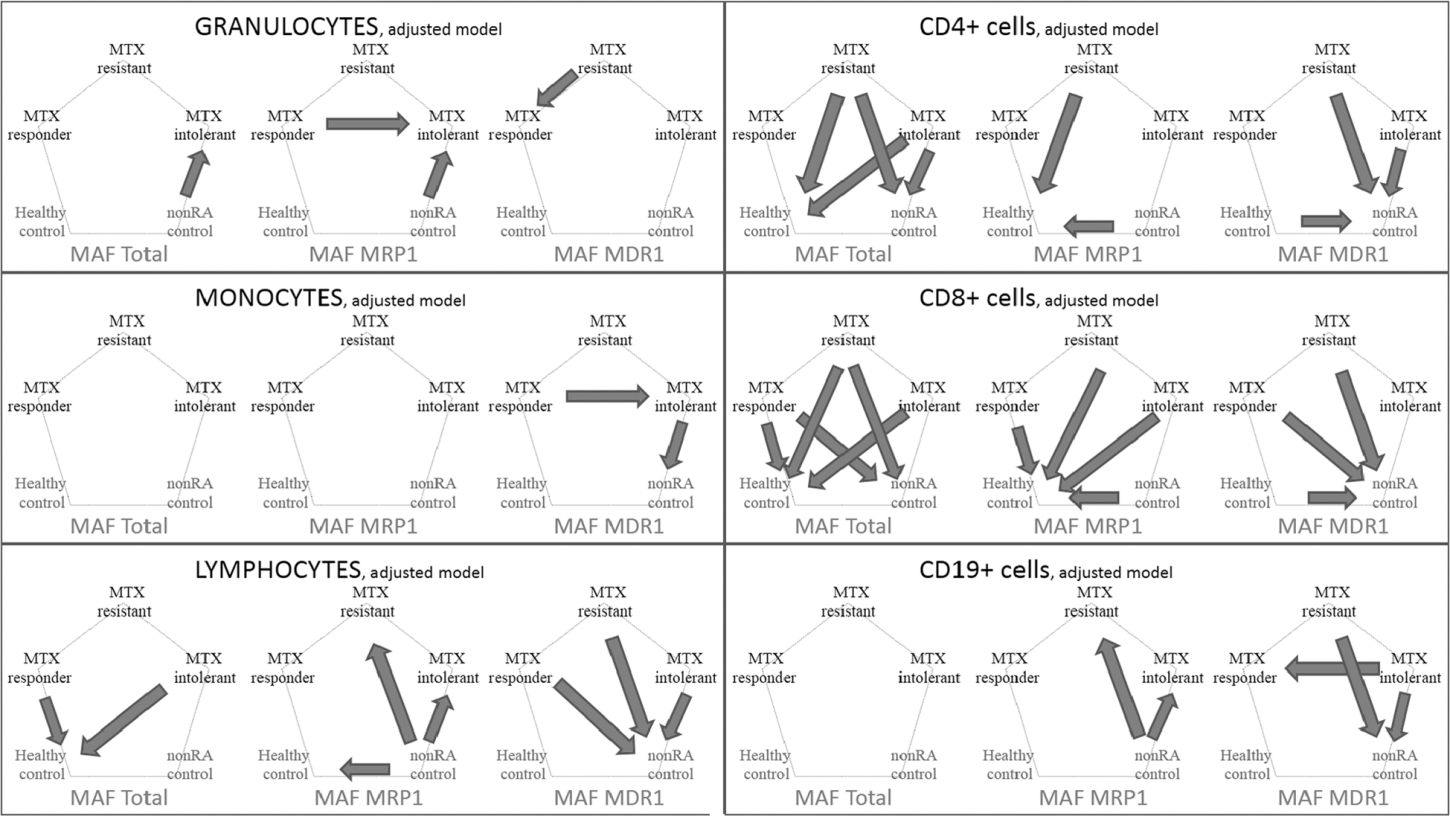
Significant differences among various patient-groups. The differences among the 5 groups are shown with arrows pointing towards the significantly higher MAF values according to the adjusted/justified model. Only significant differences with *p* < 0,05 are shown

With the adjusted model, the MAF_MDR1_ of the granulocytes was significantly higher in the MTX-responder group than the MTX-resistant group. Another finding was that the MTX-intolerant RA-group had significantly higher MAF_MRP1_ than the MTX-responders in the granulocyte subpopulation (Fig. 1). Unfortunately, the granulocyte representation was sometimes low in our samples and not all blood samples contained enough granulocytes to be subject of precise MAF determination.

With a potential clinical application, another interesting finding was the difference in MAF values in the granulocyte, monocyte and CD19+ cells between the MTX-responder and the MTX-intolerant groups. The MAF_MRP1_ of granulocytes in the MTX-intolerant group was significantly higher than that of MTX-responder RA-patients, and the MAF_MDR1_ of MTX-intolerant group was significantly higher than that of MTX-responder RA-patients in the granulocyte population. In contrast, we have found significantly lower MAF_MDR1_ of the CD19+ cells of RA-patients intolerant to MTX-therapy than those of MTX-responders.

According to our results, the most prominent difference was found between the different RA-groups and the two control groups in almost all leukocyte subgroups. Granulocytes and CD19+ cells had higher MAF_MRP1_ in the RA-groups with the adjusted model, while the other leukocyte groups have higher MAF_Total_ and MAF_MDR1_ and lower MAF_MRP1_. The differences become more pronounced in the following sequence: monocyte, lymphocyte, CD4+ cells and CD8+ cells (Fig. 1).

## Discussion

The first report on the potential role of MDR-proteins in RA found significantly higher MDR1 (P-glycoprotein) expression in steroid-treated RA-patients independently from other factors [15]. Later, in a small study by Yudoh et al. increased expression of P-Glycoprotein on Th1-cells correlated with drug resistance was observed after two month of therapy with bucillamin or sulfsalazine [16]. As DMARD therapy moved forwards, the MRP1-susbtrate MTX and the role of the MRP1 proteins have emerged in defining the response to MTX-therapy [17].

Despite our original hypothesis on the effect of functional MDR-activity on the response rate to MTX-therapy, in this study we were not able to demonstrate significant differences in the functional MDR-activities of MTX-responder and MTX-resistant RA patients, except for the higher MAF_MDR1_ of granulocytes of MTX-responders than MTX-resistants. However, we found another significant difference in the MAF values of the granulocytes: the MTX-intolerant RA-group had significantly higher MAF_MRP1_ than the MTX-responder RA-group. This difference may be useful in identifying the MTX-intolerant patients, who cannot benefit from MTX-therapy.

The MAF_MRP1_ of granulocytes and the MAF_MDR1_ of monocytes was significantly higher in the MTX-intolerant patients than in MTX-responders, while they had significantly lower MAF_MDR1_ in the CD19+ population. The combination of these three results could be clinically relevant in determining the MTX intolerant patient population.

In clinical setting: if a patient needs dose escalation of MTX and has side effects probably related to MTX, a scheduled MDR-functional assessment finding the upper mentioned triad of granulocytic MAF_MRP1_ and monocytic, CD19+ lymphocytic MAF_MDR1_ values might help in deciding to turn off ineffective dose-escalation of MTX. Titrating MTX-dosage in RA-patients is quite laborious, so disclosing patients who will not benefit from dose elevation and thus avoiding the harmful side effects of MTX-treatment might have clinical relevance in the future. Although, further studies are still needed for the clinical implementation/validation of these findings.

There are only few previous studies on RA patients investigating the role of MDR-transporters in MTX-response. Wolf et al. determined the functional activity of Reduced Folate Carrier (RFC), which transports MTX into the cells, and MRP1, which effluxes MTX out of the cells. They logically anticipated that RFC+ and MRP- patients should have better response to MTX-therapy, but instead, they observed the opposite result, since these patients had the worse response rate, while the lack or presence of both proteins was linked to significantly better therapeutic outcome [18]. In another study, they determined the mRNA-levels of FolylPolyGlutamyl Synthetase (FPGS) enzyme, which accumulates MTX into the cells, and they also found the contradictory result, such that FPGS mRNA expression is an independent predictive factor associated with poor response to MTX-therapy in RA patients [19]. Considering the rather complex metabolic and effector mechanism of MTX, more studies are needed to evaluate the effects of the various functional activities on MTX-response [20].

Our most relevant finding was the major difference between the RA and control groups. In general, the significant differences were the decreased MAF_Total_ and MAF_MDR1_ and higher MAF_MRP1_ in the RA patients compared to the control-patients calculated with or without adjustment of the effects of medicine-intake. This difference was most prominent in case of the CD8+ cells. These cells had the highest MAF-values, which is in agreement with the literature [21, 22]. Interestingly, although MTX is an MRP1 substrate, the most important and significant differences were found in MAF_Total_ and MAF_MDR1_. Hider et al. described the down regulation of MRP1 protein in PBLs by immunocytochemistry of early RA-patients after six months of MTX-therapy. They studied the MRP1 expression of 18 RA patients and 14 control persons, but they also couldn’t find any difference between the MRP1 levels of the responder and non-responder RA-patients [23]. In our study we found similar results, but we used the dynamic data of functional activity determination of MDR1 and MRP1-proteins instead of the static representation of MRP1 proteins. This, according to the earlier data, may give better insight to the significance of the MDR-proteins on treatment. Furthermore, comparing 3 RA-groups of altogether 59 RA-patients and two control groups of altogether 47 persons, we found multiple significant differences between those groups. Our results demonstrate that MTX-treatment inhibits total MDR and MDR1 functional activity in RA-patients.

## Conclusions

In conclusion, by comparing the MAF values of six leukocyte subpopulations among 3 RA-patient and 2 control groups, we have found significant difference in case of granulocytes between the MTX-responder and MTX-resistant RA-group. Our results have shown multiple differences in MAF values of the MTX-responder and MTX-intolerant RA-groups, and therefore, determining MAF in RA-patients might be useful in predicting MTX-intolerance. Titrating MTX-dosage in RA-patients is quite laborious, so disclosing patients who will not benefit from dose elevation and thus avoiding the harmful side effects of MTX-treatment may have clinical relevance in the future. Our most relevant finding was, however, the decreased functional MDR1- and MRP1-activity in MTX treated RA-patients, which might be interpreted as part of the immunosupression.

## Methods

### Patient samples

RA patients were categorized by their DAS28 improvement rate into MTX-responder (*n* = 18) and MTX-resistant (*n* = 20) groups. An additional MTX-intolerant group was also selected as RA-patients who terminated MTX-therapy (*n* = 21) due to intolerable side effects. The two control groups were composed as either hospitalized (due to other diseases or traumas) patients without RA (*n* = 20) as well as healthy, young volunteers (*n* = 27). Altogether, 106 samples were grouped in three RA-groups (*n* = 59) and two control groups (*n* = 47). All peripheral blood samples were collected with the approval of the national and local ethical committee (ETT TUKEB: Scientific and Research Ethics Committee of the Medical Research Council. Ministry of Health, Medical Research Council,Budapest, H-1051, Arany János u. 6–8, Hungary.) and all patients were involved after signing their written agreement. Blood samples were taken in 5 ml BD Vacutainer tubes with EDTA (**BD #367861)** and stored at 4 °C until transported and processed within 6 h after sampling. PBLs were isolated using HISTOPAQUE 1077 (Sigma-Aldrich, 10771).

### Calcein assay

The original calcein assay was obtained from the MultiDrugQuant Assay™ (Product No. 5 599880 083012, SOLVO Biotechnology Inc. Budaörs, Hungary, http://www.solvobiotech.com/). MDQuant Assay™ was used to determine the functional activity of MDR1 and MRP1 transporters in MAF (Multidrug Activity Factor), which is a dimensionless and standardized value calculated by the mathematical formula: MAF_Total_ = 100 x (F_Verapamil_ - F_HBSS_)/ F_Verapamil;_ MAF_MRP1_ = 100 x (F_MK571_ - F_HBSS_)/ F_Verapamil;_ MAF_MDR1_ = MAF_Total_ – MAF_MRP1_ where F stands for the average mean calcein-fluorescence values of the parallel samples. Functional MDR Activity Factor (MAF) was determined in six PBL populations (granulocytes, monocytes, lymphocytes, CD4+, CD8+, CD19+ cells).

### Immunocytochemistry

Immunostaining of the different PBL-subpopulations was performed at room temperature by incubating the blood cells in 100 μl HBSS containing either 1 μg of mouse anti-IgG1 antibody for isotype control (X0931, Dako) or 6ul anti-CD3,4,8 (CD3: C7225, CD4: C24865, CD8: C9494, Dako) or 12ul of anti-CD19 (C24825, Dako). The immunolabeling was visualized by incubation with 1 μg secondary Cy5-conjugated goat anti-mouse IgG antibody (115-175-003, Jackson Immuno Research).

### Viability staining

Non-viable cells were labeled by incubation in 200 μL HBSS containing 1 μg/ml propidium iodide (PI; 287075, Sigma-Aldrich,) for 30 min at room temperature.

### Determination of MAF-values of different white blood cell subpopulations

**Fig. 2:**
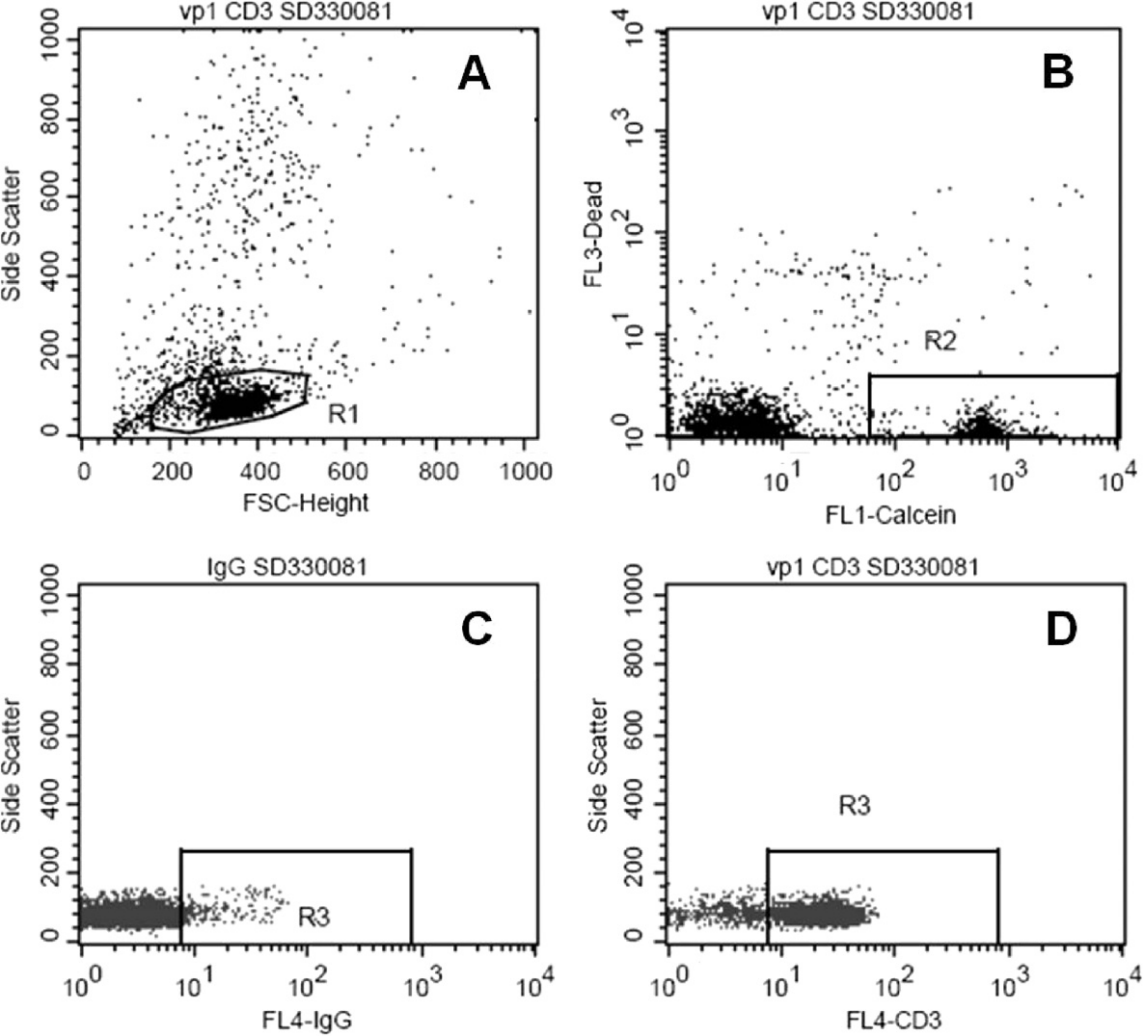
Gating out CD3+ cell population. Part **a** shows how the lymphocytes are gated with R1 in the FSC-SideSCatter dot-plot. The cells in R1 gate are shown on the Fl1-Fl3 graph of part **b**, where the viable cells are gated out by the R2 gate. Part **c** and **d** shows how the viable lymphocytes of R2 gate stain with anti-CD3 stain. R3 gate on the FL4-SSC graph is positioned to select out only the CD3+ cells

**Fig. 3:**
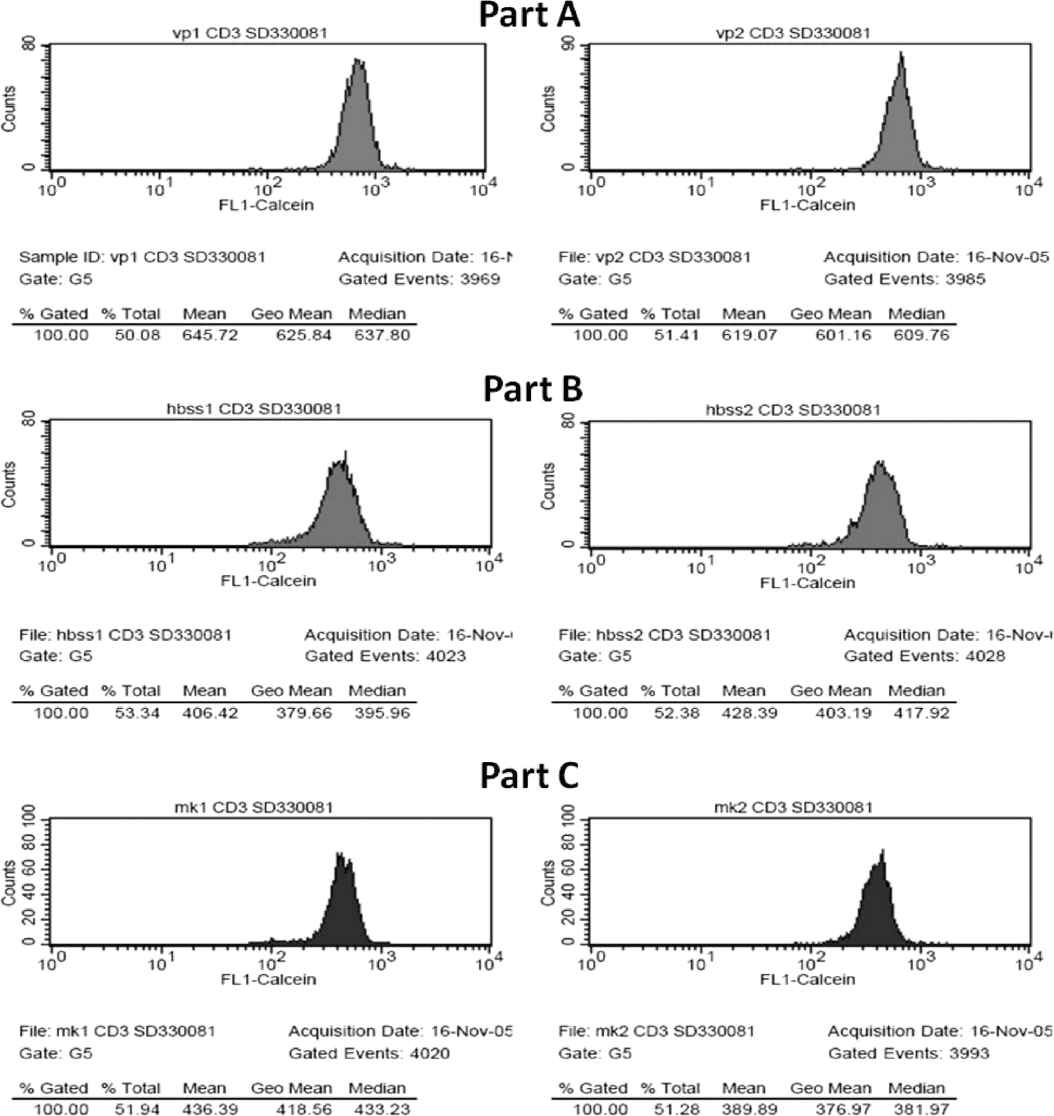
Calculating the MAF-values. This figure shows the shifts in the Fl1-Calcein-signal with the different MDR-blockers. Part **a** shows the two parallel samples treated with the MDR1 and MRP1 blocker Verapamil (Fl1-Calcein mean 645.72 and 619.07). Part **b** shows the two parallel samples of the controls incubated in HBSS (Fl1 mean 406.42 and 428.39). Part **c** shows the two parallel samples treated with the MRP1 blocker MK-571 (Fl1-Calcein 436.39 and 389.89). With these Fl1-Calcein values the different MAFs of the CD3+ lymphocytes are as follows MAF_Total_ = 34, MAF_MRP1_ = 0, MAF_MDR1_ = 34

Measurements were performed on a FacsCalibur (Becton Dickinson, Franklin Lakes, NJ, USA) flow cytometer. The viable cells were selected by the positive staining with Calcein and lack of PI staining. Granulocytes, monocytes and lymphocytes were gated on the FSC-SSC scatterplot. The CD4, CD8 and CD19 positive subpopulations were gated based on their positivity with the corresponding antibody detected at FL4 against the non-specific binding detected with IgG1 isotype control. (Gating algorithm is shown in Fig. 2.) The shifts in the calcein signal of the leukocyte subpopulations were determined with different MDR-blockers (verapamil and MK-571) compared to HBSS control. MAF_Total_, MAF_MDR1_ and MAF_MRP1_ was calculated on each subpopulations (Fig. 3).

### Statistical methods

MAF measurements were modeled using analysis of covariance (ANCOVA) with residual error stratified in patient groups to allow for unequal variances. Models were fitted using the R statistical system (R Development Core Team 2007, ISBN 3-900051-07-0), with the GLS (generalized least squares) procedure of the NLME package (R Core team, 2007, R-package version 3.1-86). Models were compared by using maximum likelihood (ML) fits, and Wald-type significance tests were performed using restricted maximum likelihood (REML) estimates. Differences were considered statistically significant below the level of *p* = 0.05. The following predictors were considered during model selection: patient group (5 levels), sample storage duration (continuous), drug doses of methotrexate, delagil, arava, medrol (continuous), administration of the same drugs (yes/no), administration of NSAIDs (yes/no). We have selected the best model using the Akaike Information Criterion (AIC) (Akaike 1974: A new look at the statistical model identification. IEEE Transactions on Automatic Control 19 (6): 716–723.) All subsets regression was not computationally feasible due to the large number of predictors, so forward and backward stepwise variable selection algorithms were applied. When the stepwise algorithm yielded a model where the same drug was included both as a continuous and binary predictor, we checked if the coefficients corresponded to a monotonous dose-effect relationship. In case of non-monotonicity we have considered the two parameters for the same drug as overfitting, dropped the model and the stepwise algorithm was restarted twice, first omitting the binary dose variant, second omitting the continuous one. When the stepwise algorithms led to different models, the one with lowest AIC value was selected.

## Abbreviations

ABC-proteins: ATP-binding cassette proteins
AIC: Akaike information criterion
Calcein-AM: calcein acetoxy methylester
DMARDs: Disease modifying anti rheumatic drugs
EDTA: Ethylene diamine tetraacetic acid
FPGS: Folylpolyglutamyl synthetase
FSC-SSC: Forward SCatter – Side SCatter dot-plot
HBSS: Hank’s balanced salt solution
MAF: Multidrug activity factor
MDR: Multidrug resistance
MDR1: MultiDrug resistance protein 1, also called P-glycoprotein-170
MRP1: Multidrug resistance related protein 1
MDQ Assay: MultiDrug quant assay^TM^
MTX: Methotrexate
NSAIDs: Non steroid anti-inflammatory drugs
PBLs: Peripheral blood leukocytes
PI: Propidium iodide
RA: Rheumatoid arthritis
RFC: Reduced folate carrier
